# Annotations

**Published:** 1893-09-30

**Authors:** 


### ANNOTATIONS.

**King's College and Public Health.**

THE Medical Faculty of King's College have recently issued a syllabus and course of instruction in Public Health, which for breadth and thoroughness leaves nothing to be desired. As General Director and Head of the Laboratory Department, the College has appointed Professor W. R. Smith, Medical Officer of Health to the London School Board. A better appointment could hardly have been made. Professor Smith, as Medical Officer to the London School Board, is necessarily in constant touch with the actual facts of public health every day of his life. The ocmbination of laboratory and outside practical work is exactly what is needed. Professor Smith, we observe also, has been called to the Bar. This, too, is as it should be. A clear and intelligent grip of the law is indispensable for a man who is appointed to conserve the public health; and such a grip can hardly be secured by the average mind except by constantly attending to the enunciation of principles and details in connexion with special training for special work. The ordinary medical officer of health must learn his law as he learns his daily duties. He will then know the one as well as the other. The syllabus issued by the College is very full and complete, and the names of the teachers inspire confidence. Professor Smith, as we have already said, is Director-General of the Laboratory. As heads and instructors in departments may be named Professor Charles Kelly, M.D., F.R.C.P., Professor Edgar Crookshank, M.B., Mr. D. Harvey Attfield, M.B., Mr. R. T. Hewlett, M.D., Professor G. H. Seeley, and many others. For senior medical students and for newly-admitted members of the medical profession who desire special instruction, or who may wish to qualify themselves, there is no course, so far as we are aware, which is more complete, if indeed there be any which is so complete, as this which King's has now provided. We advise all persons who desire instruction in public health, or in any department of it, to write for the syllabus of King's College.

**Lord Meath Physical Educator.**

THE Earl of Meath, though not a scheming man, is a man of schemes. His last new scheme is of the nature of a net to include the whole nation; it is is nothing less than the establishment of a British College of Physical Education; a college, that is to say, with its centre in London, and a branch in every considerable town in Great Britain. The College already exists in Lord Meath's brain, and he is extremely desirous of solidifying the cerebral image and putting it firmly down on solid ground. The task is by no means easy. In carrying it out his Lordship might very well be painted by a sympathetic artist as "A good man struggling with Adversity;" "Adversity" in the picture being represented by the rich British public, fiercely buttoning up its pockets, and running away at full speed, with the Earl of Meath hanging desperately on to its coat tails. In the capacity of recruiting sergeant Lord Meath has enlisted a good many persons who are very competent to speak upon and to deal with the subject of physical education. He has secured, for example, Sir Crichton Brown, M.D., and Sir Richard Webster, the late Attorney-General; Mr. Joseph Diggle, M.A., Chairman of the London School Board, and Mr. Herbert Gladstone, M.P.; Sir Spencer Wells, Dr. Thomas Savill, and Mr. Frederick Treves. These gentlemen constitute a thoroughly practical body of exports. Among more ornamental persons may be named General Lord Wolseley, the Honourable Chandos Leigh, General Moberley, General Hammersley, Colonel G. M. Onslow, and Colonel Fox. Of professional physical educators a good many have given in their adhesion to the scheme, among whom may be mentioned Mr. H. H. Burdett, of the People's Palace; Mr. Guy Campbell, of the Royal Normal College; Miss C. Long-Wilson, of the Orion; Miss K. Parry, of the St. Andrew's Club, with a good many others. For our part, we are convinced that, since everybody is now rushing from the country to live and work in the town, we must either take steps to promote the physical development of our people, or we shall shortly find such a general impairment of physique as will threaten the very existence of the nation. A National College of Physical Education is an imperative necessity: and if there were but a dozen really far seeing and practical doctors in the House of Commons they would make common cause and compel Parliament to establish such a college on a national basis and with national funds. But in the meantime Lord Meath's scheme is ready, and is only waiting for money. An excellent way of providing this money would be for four or five rich aristocrats like the Duke of Westminster to come forward with twenty or thirty thousand pounds each, and to at once establish this truly Imperial Institution; or, in order that the City rather than the West-end may have the glory, we would ask half-a-dozen of our merchant princes to find the needful funds. In the meantime associates may join the College by the payment of an annual guinea. We commend the scheme to the consideration of every grown-up man in Great Britain who desires the permanence of our Empire. The Central Office is at 92, Long Acre, W.C., for the present.

**Curiosities of Cerebration.**

WHEN medical men, as some day will be the case, shall have cast away their love for the possession of mysteries and secrets, and lawyers and judges, as will also some day be the case, shall be willing to be taught what they do not know, instead of assuming that what they are ignorant of is not knowledge, then will come a time when the world will be much wiser than it now is, and there will be a great deal more justice done all round. The *Edinburgh Medical Journal* for September, which, by the way, is an interesting and excellent number, publishes an article which, among other things, discusses the question of the effects of brain changes on speech. A patient is mentioned who suffered from what is called "Gibberish Aphasia." This poor man evidently knew as well as anybody else exactly what was going on around him. He was perfectly sane in all respects, and if his tongue would have obeyed his understanding all would have been well. But when he began to speak, nothing whatever but absolute "gibberish" would come out of his mouth. The only coherent words he could utter, and those only at times and by accident, were "If you please, sir." Another patient, who also was sane, could not even read aloud correctly from a book when the page was open before him. When asked, for example, to read aloud the passage, "It shall be in the power of the college to examine or not to examine any licentiate," he invariably read it thus: "An the be what in the temothar of the throthotodoo to majorum." This patient recovered in due time and spoke like other people. One of Trousseau's cases was decidedly amusing, at any rate to those in the secret. A visitor calls and is introduced to Madame B. Whereupon the lady rises, and with the politest of tones and gestures addresses the visitor as "Pig, Brute, Stupid fool," &c. "Madame is inviting you to take a seat," explains her medical attendant. These cases could be easily multiplied, and are by no means uncommon in ordinary medical experience. What it is desired to insist upon is that all the world, and especially those classes who have to deal with the minds and morals of their fellow-creatures, should be widely familiar with this and every other phase of human aberration. We are only at the beginning of scientific and reasonable human history; and it is highly desirable that all rational people should thoroughly comprehend the fact.

